# Molecular survey of *Cryptosporidium* spp. in ornamental birds and their sellers: implications for One Health

**DOI:** 10.1186/s12917-026-05666-7

**Published:** 2026-06-27

**Authors:** Fatemeh Kholusi, Ehsan Javanmard, Elham Kazemirad, Mohammad Ali Mohaghegh, Alireza Larifi, Mehdi Mohebali, Shahrokh Izadi

**Affiliations:** 1 Department of Medical Parasitology and Mycology, School of Public Health, Tehran University of Medical Sciences, Tehran, Iran; 2 Department of Parasitology and Mycology, School of Medicine, Shiraz University of Medical Sciences, Shiraz, Iran; 3 Department of Laboratory Sciences, School of Paramedical Sciences, Torbat Heydariyeh University of Medical Sciences, Torbat Heydariyeh, Iran; 4 Health Sciences Research Center, Torbat Heydariyeh University of Medical Sciences, Torbat Heydariyeh, Iran; 5 Department of Clinical Laboratory Sciences, School of Allied Medical Sciences, Tehran University of Medical Sciences, Tehran, Iran

**Keywords:** Zoonoses, Pet birds, Molecular epidemiology, Public health

## Abstract

Cryptosporidiosis, a disease caused by the protozoan parasite *Cryptosporidium*, can lead to prolonged and chronic diarrhea in individuals with weakened immune systems. The zoonotic characteristics of this parasite may impose notable economic challenges. This study investigates the prevalence of *Cryptosporidium* in ornamental birds and their sellers in Isfahan, the central region of Iran.

A total of 223 samples, comprising 193 bird faecal samples (86.55%) and 30 seller samples (13.45%), were collected from pet stores throughout Isfahan. The samples were microscopically examined using the modified Ziehl-Neelsen staining method. Positive samples were subsequently subjected to DNA extraction and nested PCR for *Cryptosporidium* species identification. Furthermore, a phylogenetic tree and a haplotype network were constructed to investigate the evolutionary relationships and haplotype network distribution patterns, respectively.

The infection was detected in 4.66% (9/193) of ornamental birds and 13.33% (4/30) of bird sellers. Among the infected birds, 8 (4.15%) were identified as *C. parvum* and 1 (0.52%) as *C. galli*, while all infected sellers (4/30) harboured *C. parvum*. No significant correlation was observed between demographic variables and infection rates. Notably, in two pet shops, both birds and sellers were concurrently infected, indicating a potential zoonotic transmission link.

This study reveals the presence of *Cryptosporidium* in pet birds and their sellers, emphasizing the potential for zoonotic transmission. By employing a One Health approach through phylogenetic tree construction and haplotype network analysis, the interconnectedness between human and pet bird populations is evident. As close contact between birds and their sellers may facilitate disease transmission, regular testing is highly recommended to monitor and prevent the spread of *Cryptosporidium*.

**Supplementary Information:**

The online version contains supplementary material available at https://doi.org/10.1186/s12917-026-05666-7.

## Introduction

*Cryptosporidium* spp. are protozoan parasites in the genus *Cryptosporidium* that belong to the phylum Apicomplexa [1]. *Cryptosporidium* is usually transmitted through contaminated food or water, contact with feces from infected animals or humans, and close contact with infected people or animals [2]. Due to their potential impact on humans, animals, and the environment, they are of significant concern within the One Health framework [3]. The life cycle of *Cryptosporidium* involves both asexual and sexual phases and occurs within a single host, typically humans or animals [4]. In healthy people, self-limiting diarrhea is the most frequent clinical sign and can become a significant health risk, particularly for those with immunocompromised [5]. Ornamental birds, often kept as pets and closely interacting with their sellers, serve as potential reservoirs and bridging hosts for *Cryptosporidium*, facilitating zoonotic transmission. This interconnection underscores the importance of integrated surveillance and control strategies considering the Health of animals, humans, and their shared environments [6]. Four species are specific to birds: *C. meleagridis*,* C. baileyi*,* C. galli*,* and C. ornithophilus* [7, 8].

Global and regional epidemiological data underscore the significant burden of cryptosporidiosis in both human and avian populations. In Iran, a comprehensive systematic review revealed that the pooled prevalence of human cryptosporidiosis is estimated at 4.1%, with substantially higher rates observed among immunocompromised individuals and children [9]. On the other hand, the parasite is widely distributed among domestic and avian hosts; a meta-analysis on Iranian birds reported an overall *Cryptosporidium* prevalence of approximately 7.5% [10].

The role of birds in the distribution of *Cryptosporidium* contamination requires rapid and sensitive detection methods. Traditional diagnostic tools, while useful for detection, lack the resolution necessary to distinguish among *Cryptosporidium* species and strains with zoonotic potential [11]. Molecular identification techniques, particularly polymerase chain reaction (PCR) and sequencing of genetic markers like the 18S rRNA gene, offer precise, reliable, and rapid tools to detect and characterize *Cryptosporidium* spp. from diverse hosts [12, 13]. These methods provide data to determine transmission routes, assess the risks of co-contamination between humans and birds [12]. Understanding *Cryptosporidium* infection in pet birds and their sellers through molecular epidemiology is important to strengthen collaborative efforts between veterinarians, medical professionals, environmental scientists, and pet sellers [3, 14, 15]. Such multidisciplinary approaches between veterinarians and life science professionals are critical to monitoring the emergence of human-animal shared strains, preventing disease outbreaks, and promoting sustainable practices that maintain animal health, public Health, and environmental integrity [16, 17].

In sum, the molecular identification of *Cryptosporidium* in ornamental birds and their sellers reflects the broader need for multidisciplinary strategies that recognize animal health as an integral part of human and environmental well-being [14]. This study aims the molecular characterization of *Cryptosporidium* species from ornamental birds and their sellers in Isfahan City, Iran, to better understand the role of ornamental birds as a reservoir for zoonotic cryptosporidiosis.

## Materials & methods

### Sample collection

Between February and April 2024, a cross-sectional study was conducted involving sample collection from 15 bird shops in Isfahan, Iran. To ensure the representativeness of the data, a targeted exhaustive sampling method was employed, investigating all available ornamental birds and willing sellers within the 15 licensed major shops across different geographical zones of Isfahan city, that had previously agreed to participate. The sample size was determined based on the total available population during the study period rather than a statistical formula, reflecting an exhaustive sampling approach within the participating facilities. Specific inclusion and exclusion criteria were applied: for bird sellers, inclusion criteria required individuals to be actively working in the shop for at least 6 months and to provide written informed consent, while those operating temporary or moving stalls were excluded. For ornamental birds, all available birds present in the shop during the sampling rounds were eligible for inclusion. Birds that had received antimicrobial or antiparasitic treatments within the past two weeks were excluded to avoid false-negative results. Faecal samples were collected from 193 ornamental birds and 30 bird sellers. Sampling was not limited to symptomatic or diarrheic cases; all eligible birds and sellers were included regardless of clinical status. The ornamental birds, representing different species, were housed in separate cages. Sampling was carried out in three rounds to ensure representative data and account for intermittent oocyst shedding. For each bird, demographic variables including clinical symptoms, stool consistency, breed, species, and duration of residence in the shop were recorded. Similarly, for the sellers, data such as sex, age, education level, presence of gastrointestinal disorders, stool consistency, and feeding location were documented. All data were collected and recorded separately to facilitate comprehensive analysis (Table 1). Faecal samples were collected immediately after defecation using sterile wooden applicators, transferred into clean plastic containers, and promptly transported to the laboratory at 4 °C inside a cold chain container without preservatives before microscopic and molecular analysis. Subsequently, microscopically positive fecal samples were stored at − 20 °C until downstream molecular analysis.

**Table 1:** The logistic regression results of demographic variables in birds

Demographic Variables		Number	Positive Cases	Relative Infection Rate (%)	Significance Level
Gender	Male	94	6	6.38	0.446
	Female	99	3	3.03	
Duration of Bird Maintenance in Store	Less than 6 months	40	1	2.38	0.688
	1–2 years	65	3	4.61	
	More than 3 years	88	5	5.68	
Clinical Symptoms (Diarrhea, Lethargy, Restlessness, Respiratory Disorders)	Symptomatic	88	3	3.4	0.679
	Asymptomatic	105	6	5.71	
Type of Bird Based on Order	Columbiformes	35	2	5.71	0.755
	Passeriformes	65	2	3.07	
	Psittaciformes	93	5	4.3	
Stool Consistency	Watery	93	2	4.65	0.912
	Loose	43	2	6.06	
	Soft	33	5	4.27	

### Microscopic examination

Fresh stool samples collected from individuals with diarrhea were transported to the Laboratory at Tehran Medical Sciences University for parasitological analysis. The specimens were concentrated using the formalin-ethyl acetate technique, and smears prepared from fecal pellets were examined microscopically to detect the presence of *Cryptosporidium* spp. Oocysts using a modified Ziehl-Neelsen (MZN) technique. Briefly, slides were exposed to Carbol Fuchsin for 20 min, then washed with sulfuric acid (10%) for 40 to 60 s and re-stained with methylene blue for 1 min [18]. All avian and human faecal samples that were microscopically positive for Cryptosporidium were stored at − 20 °C for downstream molecular analysis.

### DNA extraction and PCR amplification of the 18 S rRNA locus

According to the manufacturer’s instructions for the FavorPrep™ Stool DNA Extraction Kit, before starting the DNA extraction, fecal samples were washed with phosphate-buffered saline (pH 7.2) to remove contaminants. A total of 250 µL of the prepared sample was incubated with 300 µL of lysis buffer, 25 µL of proteinase K, and glass beads in a water bath at 60 °C for 30 min, with vortexing every 10 min. Nested PCR was performed to amplify the small-subunit ribosomal RNA (SSU rRNA) gene, as previously described by Power et al. [19]. The first round of PCR utilized the primer pair 18S CF2 (5′-GACATATCATTCAAGTTTCTGACC-3′) and 18S CR2 (5′-CTGAAGGAGTAAGGAACAACC-3′) to amplify a 764 bp fragment. The second round utilized internal primers 18SIF (5′-AGTGACAAGAAATAACAATACAGG-3′) and 18SIR (5′-CCTGCTTTAAGCACTCTAATTTTC-3′) to amplify a 323 bp fragment. The first PCR round was conducted under the following conditions: initial denaturation at 95 °C for 5 min; 35 cycles of 94 °C for 40 s, 55 °C for 45 s, and 72 °C for 45 s; and a final extension at 72 °C for 4 min. For the second PCR, the conditions were set to: 95 °C for 5 min; 30 cycles of 94 °C for 35 s, 57 °C for 35 s, and 72 °C for 40 s; followed by a final extension at 72 °C for 5 min. Each PCR reaction was performed in a total volume of 20 µL, containing 10 µL of 2X Ampliqon Master Mix (Ampliqon, Denmark), 1 µL of each forward and reverse primer (10 pmol/µL), 2 µL of DNA template, and 6 µL of nuclease-free deionized water. All reactions were carried out in a sterile environment to ensure precision and reliability. To analyze the amplification products, 5 µL of the second-round PCR product was loaded onto a 1.5% agarose gel containing Green Viewer in TAE buffer, alongside positive (PQ7970023) and negative (distilled water without nuclease) controls and a 50 bp DNA ladder. Electrophoresis was performed at 85 V for 30 min, and the bands were visualized and recorded using a Gel Doc system. After gel electrophoresis, positive amplicons were purified and submitted to Pishgam Biotechnology Company (Tehran, Iran) for Sanger sequencing in a forward direction using the secondary primer to determine specific species.

### Statistical analysis

The prevalence of *Cryptosporidium* spp. was calculated as the percentage of positive samples divided by the total number of samples examined, expressed with a 95% confidence interval (CI). Descriptive statistics were used to summarize the frequency and distribution of *Cryptosporidium* species among ornamental birds and sellers. Chi-square or Fisher’s exact tests were performed to evaluate the correlation between demographic variables and the presence or severity of infection, using SPSS software. A p-value of less than 0.05 was considered statistically significant.

### Haplotype network and phylogenetic analysis

The quality of the raw electropherograms was assessed, and low-quality terminal bases were trimmed using BioEdit software (version 7.2.5). The resulting consensus sequences were subjected to a BLAST search (Basic Local Alignment Search Tool) against the National Centre for Biotechnology Information (NCBI) GenBank database to identify the species and determine preliminary sequence homology. Network analysis utilizing PopART (version 1.7) (http://popart.otago.ac.nz) was performed to build linkages between *C. parvum* isolates from various sources. This method is typically used to depict the genetic relationships between and within species inferred through diverse approaches, such as the Templeton, Crandall, and Sing (TCS) genealogical method. Accordingly, the TCS network was constructed using a parsimony-based analysis of the aligned sequences to illustrate the relationships and genetic distances among the isolates [20].

Chromas and BioEdit were used to manually edit and trim each sequence. The Basic Local Alignment Search Tool (BLAST) was used to compare modified sequences with the GenBank database to identify species. All sequences generated in this study were deposited in GenBank under accession numbers (PQ813566–PQ813577). The optimal phylogenetic tree was constructed using MEGA 11 software via the Maximum Likelihood (ML) algorithm, with the best-fit nucleotide substitution model selected based on Bayesian Information Criteria (BIC). For this purpose, the Tamura 3-parameter model was utilized. Additionally, *Cryptosporidium andersoni* was selected and utilized as an outgroup to root the phylogenetic tree. Furthermore, a bootstrap analysis with 1,000 replicates was executed to evaluate the statistical reliability, robustness, and significance of the tree branches [21].

## Results

### Demographic data

Of the total 193 birds sampled, 94 were males and 99 were females. Eighty-eight (43%) birds were over three years old, 105 (54.4%) were asymptomatic, and 88 (45.6%) birds had symptomatic. The birds were from three genera: *Columbiformes*, *Passeriformes*, and *Psittaciformes* (Table 1). Additionally, all 30 bird sellers who participated in this study were male. Regarding their age distribution, 18 (60%) of the sellers were between 25 and 35 years old, while the remaining 12 (40%) were in the 35–50 age group. Gastrointestinal symptoms were assessed, revealing that 23 (76%) of the sellers were completely asymptomatic, whereas 7 (24%) presented with various digestive disorders, including diarrhea, abdominal pain, nausea, and bloating (Table 2). When correlating clinical signs between hosts from the same facility, no concurrent symptomatic infections were found; the symptomatic birds and symptomatic sellers did not originate from the same pet shops.

**Table 2:** The logistic regression results of demographic variables in sellers

Demographic Variables		Number	Positive Cases	Relative Infection Rate (%)	Significance Level
Gender	Male	30	4	13.33	0.324
	Female	0	0	0	
Age Groups	25–35	18	1	5.58	
	35–50	12	3	25	
Education Level	Illiterate and Elementary	6	1	16.66	0.364
	Secondary	15	3	20	
	University	9	0	0	
Gastrointestinal Disorders (e.g., diarrhea, abdominal pain, nausea, bloating)	Symptomatic	7	1	14.28	1
	Asymptomatic	23	3	13.04	
Stool Consistency	Watery	2	2	100	0.003
	Loose	9	1	11.11	
	Soft	13	1	7.7	
	Form	6	0	0	
Feeding and Water Source of Sellers	In the shop and using the same water source as the bird	18	2	11.11	1
	Outside the shop	12	2	16.16	

### Microscopic examination

**Fig. 1:**
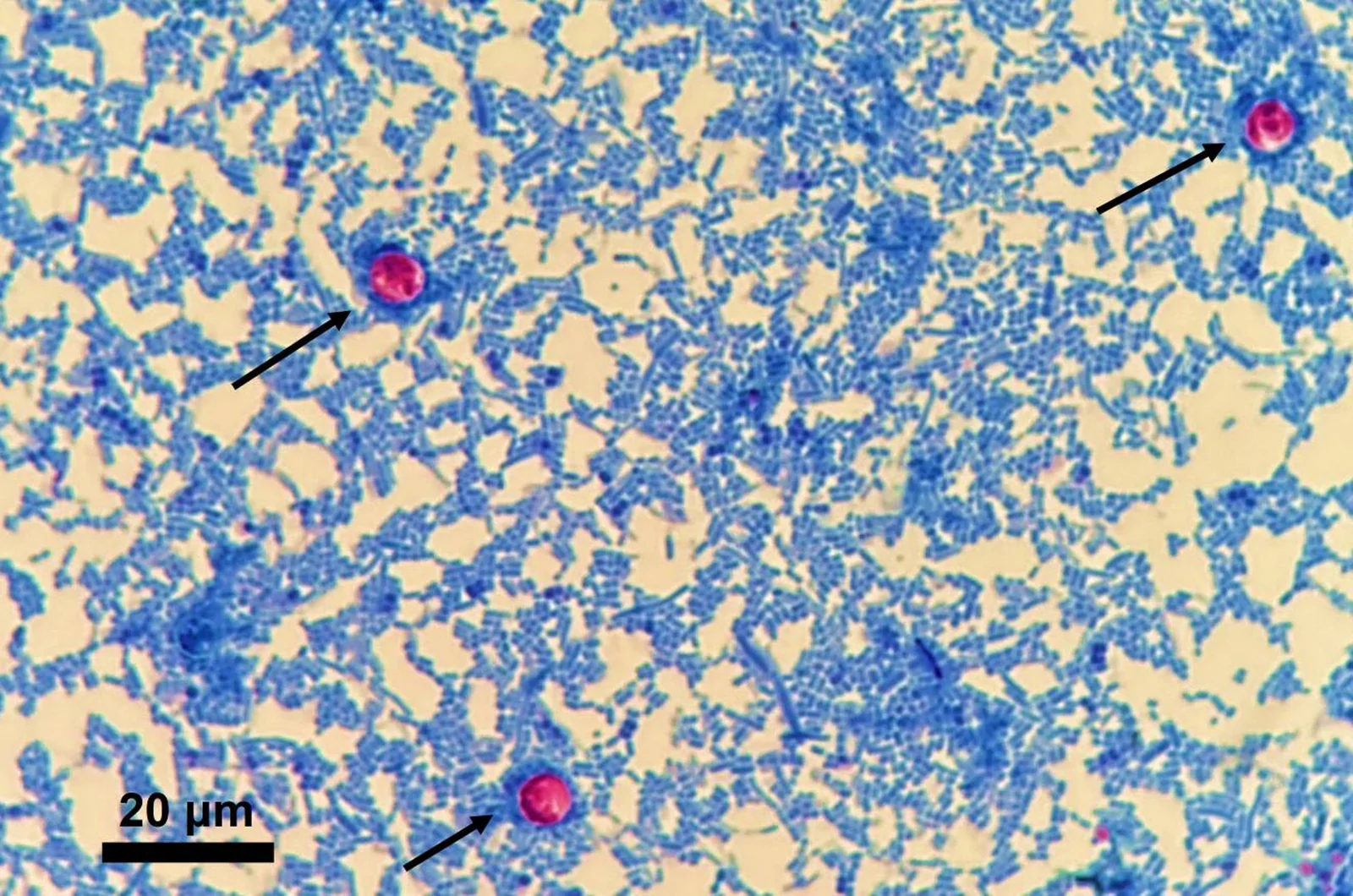
Staining of *Cryptosporidium spp*. using the modified acid-fast method in this study

A comprehensive analysis of 193 avian faecal samples revealed that 9 were found to be positive for the target pathogen. Thirty faecal samples were obtained from bird sellers revealed 4 positive samples identified through the application of acid-fast staining techniques (Fig. 1). Among the microscopically positive birds, 3 exhibited clinical symptoms (such as diarrhea, lethargy, or respiratory issues), while 6 were completely asymptomatic. For the bird sellers, 1 symptomatic individual (reporting gastrointestinal distress) and 3 asymptomatic individuals showed positive results under microscopic screening.

### Molecular analysis

The nested PCR analysis of the 18 S ribosomal gene demonstrated that 9 out of 193 ornamental birds (4.66%) were infected with *Cryptosporidium* spp., with 8 identified as *Cryptosporidium parvum* and 1 as *C. galli* (Table 3). Notably, *C. parvum*, a zoonotic species, was the predominant one. Among the 30 bird sellers, 4 individuals (13.33%) were also positive for *C. parvum* (Table 4). When correlating molecular data with clinical status, it was found that the majority of PCR-confirmed infections occurred in asymptomatic hosts. Specifically, 6 out of the 9 positive birds (5.71% of the asymptomatic group) and 3 out of the 4 positive sellers (13.04% of the asymptomatic group) did not exhibit any visible clinical signs. Only 3 birds (3.4% of the symptomatic group) and 1 seller (14.28% of the symptomatic group) were both clinically symptomatic and PCR-positive. In two pet shops, both birds and the sellers were contaminated, highlighting direct human exposure (Table 5). In Pet Shop A, an asymptomatic seller (39 years old) harbored *C. parvum* alongside two infected birds; of these, the *Nymphicus hollandicus* was asymptomatic, while the *Pyrrhura molinae* displayed clear clinical symptoms. In Pet Shop B, both the 24-year-old seller and the infected bird (*Taeniopygia guttata*) were completely asymptomatic despite being PCR-positive. The single symptomatic, *C. parvum*-positive seller (48 years old) belonged to a separate facility and had no direct link to the tracked coincidental infections. No significant correlation was found between demographic factors and infection severity, suggesting involvement of widespread environmental or collective risk factors.

**Table 3:** Taxonomic distribution and demographic characteristics of ornamental birds infected with *Cryptosporidium* spp.

Bird Order	Scientific name	Gender	*Cryptosporidium* spp	Access number
Columbiformes	*Columba livia*	Male	*C. parvum*	PQ813577
	*Columba livia*	Male	*C. parvum*	PQ813570
Passeriformes	*Acridotheres tristis*	Female	*C. galli*	PQ813576
	*Taeniopygia guttata*	Male	*C. parvum*	PQ813569
Psittaciformes	*Psittacus erithacus*	Male	*C. parvum*	PQ813568
	*Myiopsitta monachus*	Male	*C. parvum*	PV035092
	*Agapornis fischeri*	Female	*C. parvum*	PQ813573
	*Pyrrhura molinae*	Male	*C. parvum*	PQ813572
	*Nymphicus hollandicus*	Female	*C. parvum*	PQ813571

**Table 4:** Demographics of bird shop owners

Gender	Age	Symptomatic / Asymptomatic	*Cryptosporidium* spp	Access number
Male	24	Asymptomatic	*C. parvum*	PQ813574
Male	36	Asymptomatic	*C. parvum*	PQ813566
Male	48	Symptomatic	*C. parvum*	PQ813575
Male	39	Asymptomatic	*C. parvum*	PQ813567

**Table 5:** Coincidental infection of ornamental birds and sellers with *C. parvum*

		Gender	Age	Symptomatic / Asymptomatic	*Cryptosporidium* spp
Pet shop A	Sellers	Male	39	Asymptomatic	*C. parvum*
	Bird Order	*Nymphicus hollandicus* (Female)		Asymptomatic	*C. parvum*
		*Pyrrhura molinae* (Male)		Symptomatic	*C. parvum*
Pet shop B	Sellers	Male	24	Asymptomatic	*C. parvum*
	Bird Order	*Taeniopygia guttata* (Male)		Asymptomatic	*C. parvum*

### Phylogenetic analyses

A phylogenetic tree was constructed for all but one of the *Cryptosporidium*-positive samples (accession number PV035092 due to short sequence length), along with the corresponding reference strains. The phylogenetic tree placed the isolates from our study alongside animal isolates (birds and ruminants). In addition, a *C. galli* isolate identified in this study was found to be genetically linked to avian isolates from Iraq, Brazil, and China, underscoring the significance of this *Cryptosporidium* species in avian populations worldwide, including those in Iran.

**Fig. 2:**
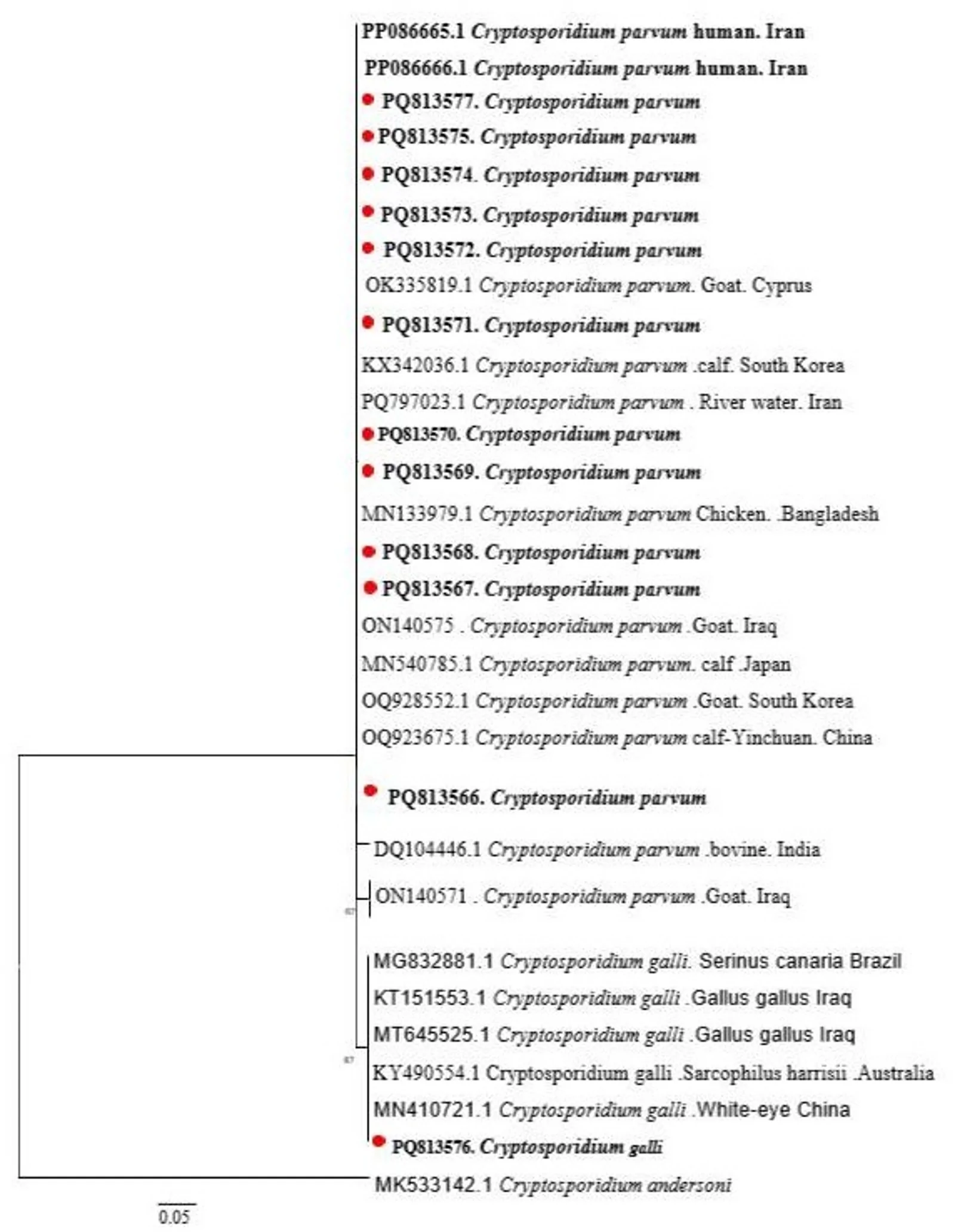
Phylogenetic tree obtained using the maximum likelihood algorithm and the Tamura 3-parameter nucleotide substitution model in MEGA 11 software. Samples from this study are marked with red circles. Bootstrap values ​​from 1000 replicates were used to assess the statistical validity of the tree

The phylogenetic tree in this study illustrates the shared nature of *C. parvum* between humans and birds, as well as the transmission cycle from animals, particularly birds, to humans in the study area (Fig. 2).

### Haplotype network analysis

**Fig. 3:**
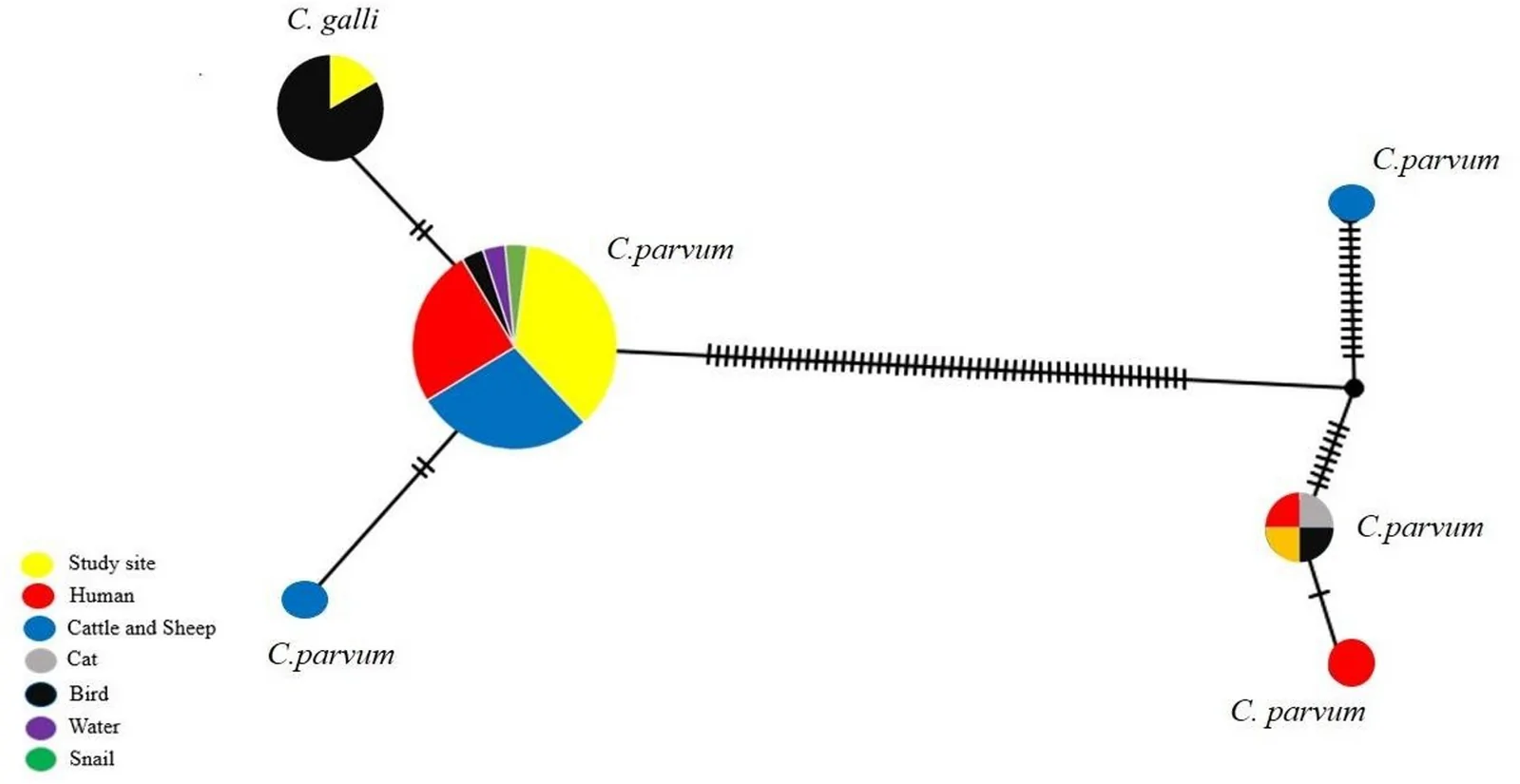
Haplotype network analysis using PopArt software (version 1.7) and based on parsimony-based analysis and TCS method, reveals the genetic relationships between *C. parvum* and *C. galli* isolates. *C. parvum* isolates collected from different sources (study site, human, cattle, sheep, bird, cat, water and snail) form a large haplotype. In contrast, *C. galli* formed a separate haplotype that is distinguished from *C. parvum* by two mutations, indicating their different evolutionary paths

Analysis of the haplotype network for *C. parvum* isolates, generated using PopArt software, revealed that these isolates clustered within a large haplotype node near animal sources, including sheep, cattle, birds, water, and snails. Conversely, *C. galli* occupied a distinct haplotype node, displaying two distinct mutation rates from *C. parvum* and was situated near other avian isolates, indicating divergent evolutionary trajectories (Fig. 3).

## Discussion

The detection of *Cryptosporidium* in birds and their sellers raises significant public health concerns, considering the zoonotic characteristics of this parasite [22]. This highlights the need for increased surveillance and implementation of effective control measures to mitigate potential transmission between birds and sellers. Based on the results of this study, the prevalence of *Cryptosporidium* parasites was estimated to be 4.66% in ornamental birds and 13.33% in their sellers. A review of studies indicates that the overall prevalence of *Cryptosporidium* spp. in Iranian birds is estimated at 7.5%. Reported prevalence rates in avian hosts have varied widely; this variation is dependent on factors such as sampling location and methodology [10]. For example, the infection rate among poultry farmers in Tabriz and its suburbs was 14%, whereas among local turkey breeders in the western region of Iran, it was 29%, which was higher than the prevalence observed in the present study [23, 24]. These prevalence differences may be due to factors such as intensive farming methods, which involve raising large numbers of birds in confined areas, resulting in crowded conditions. Additionally, specific farm management practices can create an environment that facilitates the survival and transmission of parasites. Interestingly, the prevalence of *Cryptosporidium* spp. in domestic pigeons in Isfahan was reported to be 6%, which aligns precisely with the findings of this study conducted in the same geographical area [25]. The discrepancy in prevalence between domestic pigeons and birds in this study could potentially be explained by the greater mobility and flight capabilities of domestic pigeons. This increased range allows them to cover larger distances, thereby potentially exposing them to a higher risk of infection. In addition, *Cryptosporidium* detected in bird sellers in Isfahan showed a prevalence of 16.4% in cages where lovebirds were kept, 10% in Dutch brides, and 10% in canaries. The lower prevalence of *Cryptosporidium* in our study compared to the previous research in Isfahan’s bird shops may be due to differences in sampling methods [26]. The earlier study sampled multiple cages, potentially increasing the likelihood of detecting infections from a broader population. While this study focused on individual cages, it reduced the chance of identifying positive cases. Additionally, changes in environmental conditions, bird health status, or hygiene practices between the two studies could also contribute to the observed differences in *Cryptosporidium* prevalence. Although the prevalence of *Cryptosporidium* spp. was higher in bird stores than in domestic pigeons in South Khorasan, eastern Iran, with a prevalence rate of 2.94%, this difference in prevalence may be attributed to the use of molecular tests, such as nested PCR, in this study. In contrast, the South Khorasan study employed the modified Ziehl–Neelsen staining technique [27]. Furthermore, overcrowding within pet store cages is a critical compounding factor; high-density housing induces crowding stress that compromises avian immunity, while concurrently facilitating rapid horizontal transmission of *Cryptosporidium* oocysts through close contact and fecal contamination of shared feeders and water [28].

Additionally, a study in Brazil found that the prevalence of *Cryptosporidium* in bird pet shops was 6.9%, which is higher than the prevalence reported in this study [29]. Environmental and ecological factors may influence differences in prevalence, and Further studies could clarify these differences. Statistical findings showed no significant relationship between variables and the prevalence of cryptosporidiosis (p-value > 0.05). Despite having more female birds (50.51%), the frequency of *Cryptosporidium* was higher in male birds. This matches Zhang et al.‘s study in China [30].

Collecting fecal samples from wild and domestic birds is beset with difficulties due to environmental constraints and the protection of wild animals, Stress and discomfort, and Behavioral interference. Despite all these problems, the global prevalence of *Cryptosporidium* in wild birds is 3.96% [6]. In this study, molecular testing was employed to confirm the presence of *Cryptosporidium*, which was identified through staining techniques. The process of microscopic identification of *Cryptosporidium* spp. is time-consuming, typically requiring several hours, and necessitates careful observation of microscopic specimens, which can lead to diagnostic errors [31].

In this study, molecular testing was employed to confirm the presence of *Cryptosporidium*. To improve both the accuracy and sensitivity of the detection, implementing a secondary PCR step can be highly beneficial [32]. Therefore, the use of the nested PCR method greatly helped in identifying *Cryptosporidium* species in this study, allowing for the identification of 12 cases of *C. parvum* and one case of *C. galli.* In earlier studies, the occurrence of *Cryptosporidium* among birds housed in Brazilian bird markets and pet shops was identified at a rate of 6.8%, based on 7 cases out of 103 examined [29]. Meanwhile, the prevalence observed in China ranged 3/2% to 13/4% among birds housed in markets and pet stores [33–36]. Furthermore, differences in host species composition, genetic variability of *Cryptosporidium *isolates, and the presence of species-specific transmission dynamics may further explain the discrepancies in prevalence rates reported across different geographical regions and study populations.

This study represents the first reported case of *C. galli* in Iran’s host *Acridotheres tristis*. Studies have shown that six *Cryptosporidium* species, namely *C. andersoni*, *C. parvum*, *C. meleagridis*, *C. avium*, *C. baileyi*, and *C. galli*, have been identified from *Cryptosporidium* contamination reports in avian hosts [6]. The identification of *C. galli* in this study aligns with findings from a similar study in Japan, where *C. galli* was isolated from Passeriformes [37].

Gomez et al. in Brazil and Lu et al. in China reported that *C. parvum* was the predominant species, findings that are consistent with the results of this study [29, 38]. However, in Iran, the most common species infecting various hosts is *C. parvum* [9]. Meanwhile, the predominant species identified in cancer patients in Isfahan city (central Iran) was *C. parvum*, which was geographically identical to that found in this study [39]. *C. parvum* has been detected in surface water sources and hosts such as humans and cattle in Iran, indicating that the shared transmission cycle of this species between humans and livestock remains active in Iran [40–42].

Various studies indicate *C. parvum* is the predominant species found in patients with different gastrointestinal cancers [43]. Infected patients in Finland demonstrated a higher prevalence of *C. parvum* than other *Cryptosporidium* species. Notably, individuals infected with *C.parvum* often reported prior contact with domestic animals, particularly cattle [44]. *C. parvum* varies in two different regions, Iran and Finland, but the relationship with animals is prominent in both studies. In the current study, birds play a significant role in transmission, whereas in Finland, the relationship is more closely tied to cattle. These differences may be related to differences in climate, habitat, and type of human contact with animals [45, 46]. A key finding is that transmission patterns and Host adaptation types can vary across different regions [47]. The present study emphasizes that effective prevention and control strategies should be regional and based on a precise understanding of the sources and vectors of infection, as transmission patterns differ in each region.

The phylogenetic tree image in this study shows that *C. parvum* isolates isolated from Ornamental bird sellers and birds in this study fall into a single clade. This suggests that there is a risk of transmission depending on the occupation of individuals. From One Health’s perspective, the placement of *C. parvum* in the same clade with other *C. parvum* found in different hosts in various countries suggests that it has the potential for transmission between animals, birds, and humans, which could pose a threat to human health.

Haplotype network analysis of *C. parvum* and *C. galii* across birds, sheep, cattle, and humans clearly illustrates the genetic interconnectedness among these hosts. The common haplotypes observed across different species highlight the low genetic differentiation, suggesting ongoing transmission and shared sources of contamination. This genetic overlap highlights the zoonotic potential of *C. parvum*, underscoring the complex interconnection between animals and humans in disease ecology. The shared haplotypes imply that efforts to reduce contamination in one host can have a beneficial impact on others, advocating for integrated surveillance, shared diagnostic tools, and joint intervention strategies. Several studies have demonstrated the presence of shared haplotypes among *Cryptosporidium* spp. in both domestic and wild animals. The network based on 18S rRNA haplotypes showed a similar pattern to the phylogenetic tree, consistent with findings from a study in Jordan [48].

Overall, the haplotype network plot indicates that genetic and geographic data from various hosts (humans, wild, and domestic animals) are suitable for understanding the distribution of *Cryptosporidium*. These findings emphasize the importance of the One Health approach [3].

Overall, understanding the transmission of *Cryptosporidium* in pet and commercial poultry sellers requires a detailed cryptography similar to that previously likened to deciphering the Rosetta Stone [49]. This highlights the profound importance of localized, small-scale molecular epidemiological surveys in providing the missing baseline data necessary for the practical application of the One Health framework. By bridging the gap between human occupational health and avian reservoirs, such studies lay the groundwork for comprehensive surveillance strategies required to mitigate zoonotic risks globally [6]. Several limitations in this study should be noted. First, the relatively small sample size of both ornamental birds and sellers may restrict the generalizability of our epidemiological findings. Second, the absence of gp60 gene genotyping for *C. parvum* isolates stands as a limitation. Consequently, future large-scale studies with higher sample sizes and genotyping resolution are required to precisely elucidate the micro-transmission dynamics of *Cryptosporidium* spp. under the One Health framework.

## Conclusion

In this comprehensive analysis of 193 fecal samples from ornamental birds, molecular detection methods identified nine positive samples: 8 were attributed to *C. parvum* and 1 to *C. galli*. Further examination of 30 fecal samples from ornamental bird sellers revealed four positive samples of *C. parvum*. The phylogenetic tree analysis and haplotype network visualization in this study demonstrate a genetic and evolutionary resemblance between the isolates in this research and those found in other hosts across various investigations. These findings suggest an active transmission cycle of *Cryptosporidium *spp. between ornamental birds and their sellers, highlighting the need for integrated surveillance and control strategies within a One Health framework to mitigate the risk of zoonotic transmission. 

## Supplementary Information

Supplementary Material 1.

## Data Availability

All data generated during this study are publicly available.
